# Effects of *Lactiplantibacillus plantarum* LPJZ-658 Supplementation on the Production, Meat Quality, Intestinal Morphology, and Cecal Microbiota of Broilers Chickens

**DOI:** 10.3390/microorganisms11061549

**Published:** 2023-06-10

**Authors:** Liming Liu, Letian Li, Chunhua Li, Haiyang Wang, Xiufeng Zhang, Qingdan Ren, Heping Zhang, Ningyi Jin, Chang Li, Cuiqing Zhao

**Affiliations:** 1 College of Animal Science and Technology, Jilin Agricultural Science and Technology University, Jilin 132101, China; aliuliming1984@126.com (L.L.); chunhuali2009@sina.com (C.L.); jlnkwanghaiyang@sina.com (H.W.); dwyxzxf@163.com (X.Z.); 2 Research Unit of Key Technologies for Prevention and Control of Virus Zoonoses, Chinese Academy of Medical Sciences, Changchun Veterinary Research Institute, Chinese Academy of Agricultural Sciences, Changchun 130122, China; letian823@163.com (L.L.); ningyik@126.com (N.J.); lichang78@163.com (C.L.); 3 Jilin Provincial Animal Husbandry General Station, Changchun 130062, China; qingdan_ren@163.com; 4 Department of Food Science and Engineering, Inner Mongolia Agricultural University, Huhhot 010010, China; hepingdd@vip.sina.com

**Keywords:** broiler, *Lactiplantibacillus plantarum*, LPJZ-658, growth production, meat quality, intestinal morphology, cecal microbiota

## Abstract

This study aimed to investigate the effects of *L. plantarum* LPJZ-658 on the production, meat quality, intestinal morphology, and cecal microbiota of broilers. White-feathered broilers (1 day old, n = 600) were randomly assigned to two groups and raised for six weeks. The individuals in the LPJZ-658 group were supplemented with 2.6 × 10^9^ cfu/g LPJZ-658. The growth performance, meat quality, intestinal epithelium morphology, and cecal microbiota were observed. The results showed that the average daily gain, average daily feed intake, and feed conversion ratio of broilers in the LPJZ-658 group were significantly improved. In addition, the LPJZ-658 groups had a higher thigh muscle (TM) yield, TM color, TM_pH24h_, breast muscle (BM) pH_24h_, and BM color_24h_, while the BM cooking loss was significantly lower than the CON group. Moreover, supplementation with LPJZ-658 increased ileum and cecum length, duodenum and ileum villus height, and ileum villus height/crypt depth ratio. Furthermore, 16S rRNA sequencing revealed the dietary LPJZ-658 supplementation modulated the diversity and composition of cecal microflora. At the phylum level, the relative abundances of Proteobacteria, Actinobacteria, Verrucomicrobiota, and Acidobacteriota were significantly higher. In addition, LPJZ-658 substantially decreased the genus relative abundances of Streptococcus, Veillonella, Neisseria, and Haemophilus compared with the CON group and facilitated the growth and colonization of beneficial cecal bacteria, such as OBacteroides, Phascolarctobacterium, Bacillus, and Akkermansia. It was concluded that LPJZ-658 supplementation significantly increased growth production, improved meat quality and intestinal status, and modulated the intestinal microbiota in the broilers.

## 1. Introduction

In the livestock industry, commercial broiler production has developed rapidly due to its short production cycle, excellent carcass traits, and high feed conversion rate. However, broiler chickens are is susceptible to adverse stimuli from the external environment, resulting in poor intestinal health and growth performance [1]. Since they were first developed, antibiotics have been used as growth promoters to improve feed conversion and growth performance. Antibiotics have been essential in treating infectious diseases and reducing mortality [2]. Approximately two-thirds of the antibiotics administered yearly are used in agriculture, particularly livestock. Antibiotics have been commonly employed to enhance the health of poultry production systems in several parts of the world. However, the excessive application of antibiotics may lead to antibiotic resistance among bacteria [3,4]. Recently, residual antibiotics in livestock products have received considerable attention. Bacterial resistance genes may be transmitted through multiple routes, leading to environmental pollution [5]. The addition of antibiotics to animal fodder has been banned in the European Union since 2006 and in China since 2020 [5]. This policy has created new challenges for the animal industry. Thus, it is imperative to seek novel alternatives for antibiotics in poultry feeds [6].

In recent years, researchers have discovered numerous substances, such as probiotics, prebiotics, and plant extracts, that have been reported to play a part in substituting antibiotics [7,8,9]. Probiotics are living microorganisms which benefit the host’s health by improving its nutritional and intestinal microbial balance [10]. It was shown in previous studies that dietary supplementation with probiotics can improve growth performance by enhancing digestive tract barrier functions, regulating intestinal microbiota, and increasing the levels of digestive enzymes [11]. Lactic acid bacteria (LAB) are one of the most well-known groups of probiotics. It has been verified in previous studies that LAB can improve growth performance [12], modulate the intestinal microbiome, and increase resistance against infection by enteric diseases in poultry [13,14,15]. *Lactiplantibacillus plantarum* (*L. plantarum*) strains have proven to be valuable species for developing probiotics. Research has identified that dietary *Lactiplantibacillus plantarum* added into broilers’ feeds are likely to promote growth performance through improved immunity and intestinal health and the regulation of intestinal flora [16,17,18].

*L. plantarum* LPJZ-658 (CGMCC No. 22908) was previously isolated from natural fermented dairy products by our research group. As a newly discovered strain, we demonstrated the effects of *L. plantarum* LPJZ-658 on promoting the laying ability of hens. Nonetheless, whether LPJZ-658 can enhance the growth performance of broilers remains unclear. Therefore, the purpose of this study is to investigate the effects of LPJZ-658 on the production performance, carcass traits, meat quality, intestinal status, and cecal microbiota of broilers by adding LPJZ-658 to their diet and to provide guidance on the usage of LPJZ-658 in the broiler industry.

## 2. Materials and Methods

### 2.1. Probiotic and Ethical Approval

We isolated the *L. plantarum* strain LPJZ-658 and stored it at Tianshu Yaoyuan (Tianjin) Biotechnology Co., Ltd. (Tianjin, China). The experimental protocols used in this experiment, including animal care and use, were reviewed and approved by the Animal Care and Use Ethics Committee of Jilin Agricultural Science and Technology University (Jilin, China).

### 2.2. Experimental Settings

Healthy mixed-sex broilers (white feather broilers, one day old, n = 600) with the same initial weight were randomly assigned to two treatment groups: basal diet (CON group) and basal diet containing 2.6 × 10^9^ cfu/g LPJZ-658 (LPJZ-658 group). Each group had 30 replicates and 10 broilers in each replicate. The feed ingredients and nutrient composition of the basal diets are listed in Table 1. The animal experiments were approved and conducted according to the Animal Care and Use Committee of the Jilin Agricultural Science and Technology University guidelines.

### 2.3. Growth Performance

The average daily feed intake (ADFI) and average daily body weight gain (ADG) were recorded. One broiler from each replicate was selected and weighed individually on days 1 and 42. The feed consumed for each broiler chick was monitored weekly. The ADFI, ADG, and feed conversion ratio (FCR; feed consumed/weight gain) were measured from day 1 to 42.

### 2.4. Sample Collection and Carcass Traits

After fasting for 12 h before slaughter, one broiler (42 days old) from each replicate was chosen and weighed. Blood was collected by heart puncture, and then the broilers were euthanized using a ventral neck cut and partial neck slicing using a neck cutter, followed by cervical dislocation. Intramuscular fat width and subcutaneous fat thickness were determined using a Vernier caliper. After removing the feathers and blood, the carcass weight was measured. The half-eviscerated weight was assessed by eliminating all the viscera except for the heart, liver, stomach, kidneys, and lungs. The viscera were then removed and weighed to estimate the eviscerated weight. The carcass yield, half-eviscerated yield, eviscerated yield, and abdominal fat yield were expressed as percentages of the live weight. At the same time, the left breast muscle (BM) and thigh muscle (TM) were isolated and weighed to calculate the muscle yields according to the eviscerated weight (%). The yields of abdominal fat were also determined according to the eviscerated weight (%). Subsequently, the left pectoralis and TMs were obtained and kept at 4 °C to determine pH value, drip loss (DL), and meat color. Parts of the right pectoralis and TMs were also isolated to determine cooking loss (CL). At the same time, the remaining parts were immediately frozen and kept at −20 °C for subsequent analysis. The length of the small intestines (cecum, ileum, jejunum, and duodenum) was also determined.

### 2.5. Evaluation of Meat Quality

Meat quality analysis (e.g., pH, meat color, DL postmortem, shear force, and CL) was conducted on the BM or TM. Forty-five minutes and twenty-four hours postmortem, a pH meter (PH-STAR, Matthaus, Germany) was used to measure the pH values. The pH probe was inserted into the muscle at a depth of 1 cm with constant rinsing with deionized water between the specimens. The measurements were performed in triplicate. After slaughter, the meat color was determined three times at three different positions surrounding the muscles using a colorimeter (OPTO-STAR, Matthaus, Germany) at 45 min and 24 h postmortem.

The left BMs were employed to determine the 24 h and 48 h DL postmortem through a suspension method. The muscles were weighed, suspended by a hook and line, transferred into a plastic bag, and kept at 4 °C for 48 h. The muscles were weighed again to determine DL for 24 and 48 h. The DL was determined as follows:

DL (%) = [(first weight − last weight)/first weight] × 100%.

BMs (approximately 100 g) were stored at 4 °C. CL was calculated 24 h after slaughter. Muscles were wrapped with laminated paper, weighed, and transferred into a glass beaker, followed by heating in a water bath at 75 °C for 20 min. After cooling to room temperature, the cooked samples were blotted dry. We weighed the muscles again to determine CL using the following equation:

The CL (%) = [(first weight − cooked weight)/first weight] × 100%.

After CL was measured, the BMs were used to measure shear force. After cutting into three strips of 0.25 cm (thickness) × 1 cm (width) × 2.5 cm (length), the samples were sheared perpendicular to the muscle fiber with a digital meat tenderness meter (C-LM3B, TENOVO, Shandong, China). The crosshead speed was 5 mm/s. The maximum shear force was measured for each sample, and the values (Newton force) of five cores were averaged.

### 2.6. Evaluation of Antioxidant Parameters in Muscles

After homogenizing 0.1 g muscles (1:9, *w*/*v*) with 0.9% sodium chloride buffer using a SCIENTZ-48L homogenizer (Ningbo Scientz Biotechnology, Zhejiang, China), the samples were centrifuged at 4000× *g* for 15 min at 4 °C. The supernatant was subjected to the measurement of malondialdehyde (MDA) concentrations. The superoxide dismutase (SOD) levels were assessed using corresponding diagnostic kits (Nanjing Jiancheng Institute of Bioengineering, Nanjing, China).

### 2.7. Intestinal Morphology Analysis

The intestinal segments ileum (midpoint from Meckel’s diverticulum to the ileocecal junction), jejunum (midpoint from the pancreatic duct to Meckel’s diverticulum), and duodenum (the midsection of the ascendant loop) were rinsed with cold phosphate buffer saline (PBS). Then, they were fixed in paraformaldehyde (10%) and embedded in paraffin. Next, the slides were stained with hematoxylin and eosin (H&E). Five crypts and microvilli were randomly chosen from each segment to examine VH and CD.

### 2.8. Blood Indicators

The blood of broilers was harvested by cardiac puncture, and the serum samples were centrifuged and kept at −70 °C. Serum IgM, IgG, and IgA levels were determined with a chicken ELISA kit (Nanjing Jiancheng Institute of Bioengineering).

### 2.9. Cecum Microflora Analysis

Broiler chicken cecal contents were collected and stored at −80 °C. The samples were sent to Novogene Co. (Beijing, China) for 16S rRNA sequencing under dry ice preservation. Briefly, total genome DNA from samples was extracted using the CTAB method. According to the concentration, DNA was diluted to 1 ng/µL using sterile water. V3 and V4 hypervariable regions of 16S rRNA were selected for generating amplicons and the following taxonomy analysis. DNA libraries were constructed using the TruSeq DNA PCR-free Sample Preparation Kit, and the library was sequenced on an Illumina NovaSeq platform. Paired-end reads were merged using FLASH (V1.2.7, http://ccb.jhu.edu/software/FLASH/, accessed on 17 November 2022) [19]. Quality filtering on the raw tags was performed according to the QIIME (V1.9.1, http://qiime.org/scripts/split_libraries_fastq.html, accessed on 17 November 2022) quality-controlled process [20]. Sequence analyses were performed using Uparse software (Uparse v7.0.1001, http://drive5.com/uparse/, accessed on 17 November 2022) [21]. Additionally, dilution curves, relative abundance of species, principal coordinate analysis (PCoA), and LDA effect size (LEfSE) analysis were performed in R software (Version 2.15.3).

### 2.10. Data Analysis

All data were subjected to the paired *t*-test using GraphPad Prism 7. Results were expressed as means ± standard error of mean (SEM), and the differences were deemed significant at *p* < 0.05 and extremely significant at *p* < 0.01.

## 3. Results

### 3.1. Growth Performance

The effects of LPJZ-658 on the growth performance of broilers at 42 days of age are shown in Table 2. The ADG (*p* < 0.01) and ADFI (*p* < 0.05) during the experimental period for broiler chickens in the LPJZ-658 group were significantly higher than those in the CON groups. Compared to the CON group, the FCR was significantly lower in the LPJZ-658 group from 1 to 42 days of age.

### 3.2. Carcass Characteristics and Immune Organ Index

The effects of LPJZ-658 on carcass characteristics and the immune organ index of broilers at 42 days of age are shown in Table 3. There were no significant differences in carcass yield, half-eviscerated yield, eviscerated yield, and abdominal fat yield among the broilers in the different groups (*p* > 0.05). The liver yield of broilers in the LPJZ-658 group was significantly reduced (*p* = 0.001) by the LPJZ-658 treatment. The breast muscle yield did not show significant differences between the CON and LPJZ-658 groups. However, LPJZ-658 treatment significantly increased the thigh muscle yield of broilers in the LPJZ-658 group compared to the controls (*p* < 0.01). However, the subcutaneous fat thickness and intermuscular fat width (*p* > 0.05) in each group showed no remarkable differences. Additionally, compared to the CON group, the spleen, thymus, and bursa index of broilers had no profound differences between the groups (*p* > 0.05).

### 3.3. Meat Quality Parameters

The effects of LPJZ-658 on the meat quality traits of broilers at 42 days of age are demonstrated in Table 4. Supplementation with LPJZ-658 improved the pH_24h_ (*p* < 0.05) and meat color_24h_ (*p* < 0.01) but decreased the cooking loss in the breast meat compared to the CON group. In addition, no apparent differences (*p* > 0.05) in breast meat pH_45min_, meat color_45min_, drip loss, and shear force were found across two groups. Except for thigh meat pH_45min_ (*p* > 0.05), there were no significant differences between the CON and LPJZ-658 groups. Additionally, compared with the CON group, LPJZ-658 supplementation significantly increased the thigh meat pH_24h_ (*p* < 0.05), meat color_45min_ (*p* < 0.001), and meat color_24h_ (*p* < 0.01). The effects of LPJZ-658 on the muscle antioxidant capacity of 42-day-old broilers are shown in Table S1. The results revealed that no significant differences were found in MDA concentrations (*p* > 0.05) and SOD activity (*p* > 0.05) in the BM or TM between the CON and LPJZ-658 groups.

### 3.4. Small Intestine Development and Morphology

**Figure 1:**
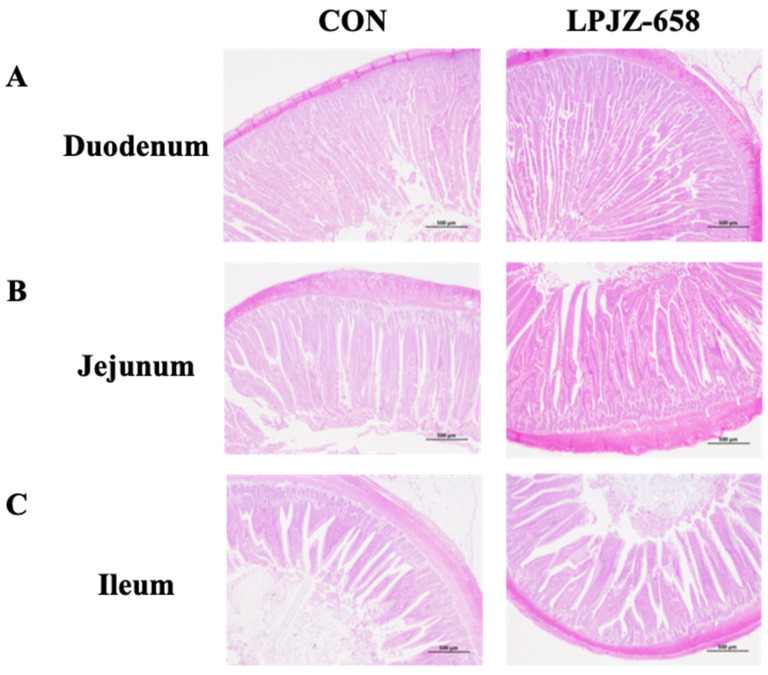
Effects of LPJZ-658 treatment on small intestine morphology in broilers: (**A**) duodenum microvillus morphology of broilers in different treatment groups; (**B**) jejunum microvillus morphology of broilers in different treatment groups; (**C**) ileum microvillus morphology of broilers fed different treatments. Scale bar, 500 µm.

As exhibited in Table 5, the results show that LPJZ-658 significantly increased the ileum (*p* < 0.05) and cecum (*p* < 0.05) length of 42-day-old broilers. There were no significant differences between duodenum and jejunum length (*p* > 0.05) among the two groups. We further evaluated the effects of LPJZ-658 on the intestine morphology in broilers. The mean values for the histomorphological changes in the broilers of the different groups are summarized in Table 6 and Figure 1. Broilers fed a diet containing LPJZ-658 exhibited greater duodenum villus height than the CON group (*p* < 0.01). However, no significant changes (*p* > 0.05) were observed for duodenum crypt depth and villus height/crypt depth (VH/CD) ratio in the broilers in the LPJZ-658 group compared with the CON group. Additionally, compared with the CON group, the ileum villus height (*p* < 0.001) and VH/CD ratio (*p* < 0.05) of broilers in the LPJZ-658 group were significantly increased by LPJZ-658 treatment. However, there were no significant differences in jejunum villus height, crypt depth, and VH/CD ratio.

### 3.5. Cecal Microbiota Analysis

**Figure 2:**
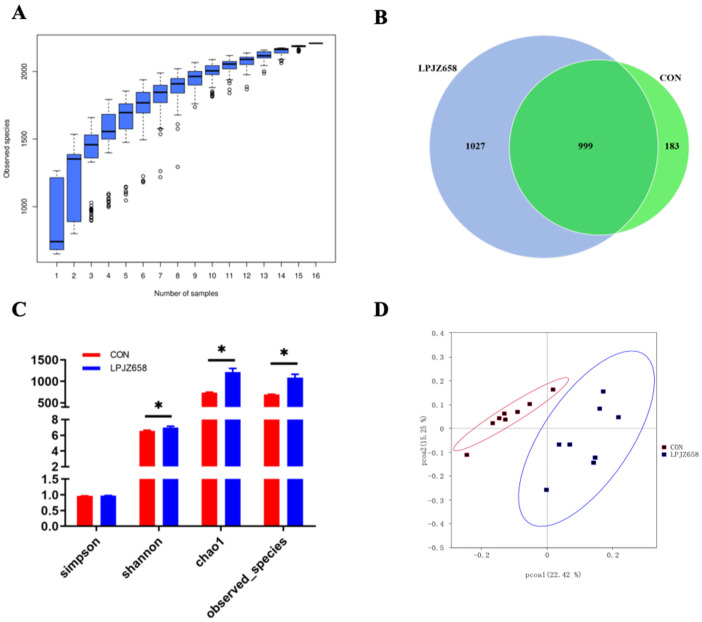
The effect of dietary supplementation with LPJZ658 on the composition of cecal microbiota: (**A**) the species accumulation boxplot; (**B**) Venn diagram; (**C**) alpha diversity indices; (**D**) principal coordinate analysis (PCoA) based on Bray–Curtis distance. Data are expressed as the means ± SEM (* *p* < 0.05).

The species accumulation boxplot (Figure 2A) tends to flatten out as the sample size increases up to 16, which indicates that our sample size was sufficient for subsequent analysis and to estimate the species richness of the samples. The Venn diagram (Figure 2B) generated after OTUs clustering with 97% homologous labels of all samples revealed that the CON and LPJZ-658 groups contained 1182 and 2026 OTUs, respectively. The number of OTUs shared by the two groups amounted to 999, whereas the number of OTUs unique to the CON group was 183 and the number unique to LPJZ-658 group was 1027. The results of the alpha diversity analysis (Figure 2C) indicated that the Shannon index, observed species, and chao1 in the LPJZ-658 group were significantly higher than in the CON group. Regarding the beta diversity of the cecum microbial community, the principal coordinate analysis (PCoA) profile of the Bray–Curtis distance (Figure 2D) demonstrated that significant discrepancies existed between the microbial communities of each group.

**Figure 3:**
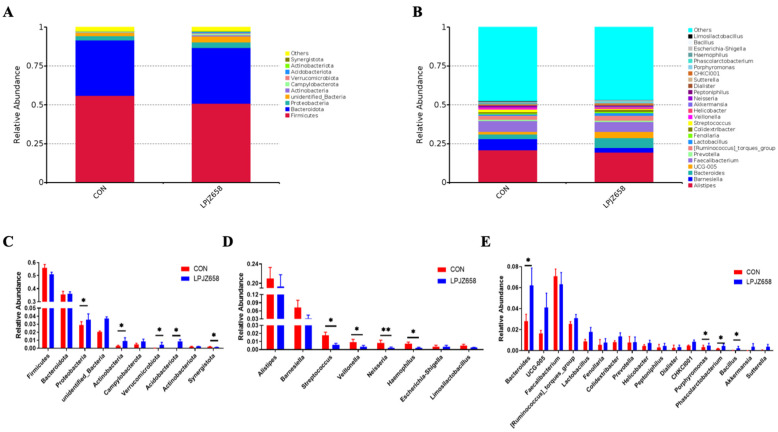
The effect of dietary supplementation with various levels of isoleucine (Ile) on the composition of cecal microbiota: (**A**) phylum level; (**B**) genus level; (**C**) the differential microbiota at the phylum level; (**D**,**E**) the differential microbiota at the genus level. Data are expressed as the means ± SEM (* *p* < 0.05 and ** *p* < 0.01).

The histograms representing the gut microbiota community structure displayed the microbial species and relative abundances of each group. Considering the bacterial composition at the phylum level, the top 10 phyla are shown in Figure 3A. Compared with the CON group, the relative abundances of Proteobacteria, Actinobacteria, Verrucomicrobiota, and Acidobacteriota were significantly elevated in the LPJZ-658 group at the phylum level, whereas the relative abundances of Synergistota were markedly decreased (Figure 3C). The relative abundances of broiler chickens’ cecal microbiota at the genus level (top 25) are shown in Figure 3B, and microorganisms with differences at the genus level are shown in Figure 3D,E. The results indicate that the relative abundances of Streptococcus, Veillonella, Neisseria, and Haemophilus were remarkably decreased in the LPJZ-658 group compared to the CON group (Figure 3D). In addition, compared with the CON group, the relative abundances of Bacteroides, Phascolarctobacterium, Bacillus, and Akkermansia were significantly elevated in the LPJZ-658 group (Figure 3E).

**Figure 4:**
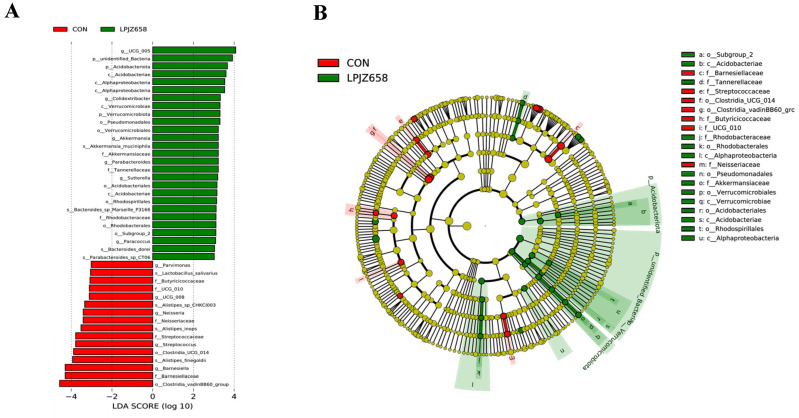
The linear discriminant analysis effect size (LEfSe) analysis of differential microbial taxa: (**A**) distribution histogram of linear discriminant analysis (LDA) values (LDA score = 3); (**B**) evolutionary branching diagram.

LEfSe analysis was used to identify the most relevant taxa responsible for the differences between the CON group and the LPJZ-658 group. An LDA cutoff score of three was used to estimate the discriminatory impact of each community on the phylogenetic distribution. The LEfSe analysis (Figure 4A,B) showed that UCG_005 in the LPJZ-658 group and Clostridia_vadinBB60_group in the CON group contributed to the differences in cecal microbiota.

## 4. Discussion

The broiler is a fast-growing and commonly farmed species in the livestock sector. The broiler industry has recently transitioned into antibiotic-free production, and viable antibiotic alternatives are necessary. Probiotics have become another potential alternative to replace antimicrobials for large-scale broiler production by improving the growth performance and general health of birds [22].

*L. plantarum* has the qualified presumption of safety (QPS) status from the European Food Safety Authorities (EFSA) and has the generally recognized as safe (GRAS) status from the US Food and Drug Administration (US FDA) [23]. Moreover, *L. plantarum* has a long history of food application [24]. *L. plantarum* strains have been shown to be valuable for developing probiotics, and isolation from fermented foods is one of their primary sources [25]. LPJZ-658, an *L. plantarum* strain we isolated from natural fermented dairy products, has been used to study the performance of laying hens with surprising results, with which it has been proven to be safe. Therefore, the purpose of current study was conducted to evaluate the effects of LPJZ-658 on the growth performance, meat quality, intestinal morphology, and cecal microflora in broilers, finally providing reference and theoretical basis for the application of LPJZ-658 in poultry.

Enhanced growth performance in broiler chickens fed dietary probiotics has been shown in previous studies [26,27]. The performance of a domestic animal is directly affected by its feed intake [28]. During the entire period of this study, broilers fed with LPJZ-658 had higher ADG and ADFI and lowered feed/gain ratio than those fed with a basal diet only. *L. plantarum* can improve intestinal health and enhance digestion and absorption [29]. Adequate health, digestion, and absorption are related to increased feed intake and growth performance [30]. It appears that LPJZ-658 could enhance the growth performance of broilers by improving the bioavailability of feed. According to this finding, LPJZ-658 supplementation was shown to have good potential as an alternative to antibiotics for improving broiler growth.

Carcass traits constitute a significant indicator in evaluating poultry production and meat quality. The protective effect of dietary probiotics on the carcass features of broilers has been demonstrated in previous studies [31,32,33]. However, generally, the inclusion of probiotics did not benefit the percentage of carcass yield and organ weight [34,35,36]. Similarly, in the present study, the carcass yield, half-eviscerated yield, and eviscerated yield of broilers in this study were not affected by the treatment with LPJZ-658. Even so, we also observed that the supplementation of LPJZ-658 increased the TM yield of broilers, which might indicate a beneficial effect of LPJZ-658 on protein metabolism. A consistent result has been reported in another study. Ashayerizadeh et al. showed that mixing prebiotics and probiotics could increase the TM ratio [37].

Studies on the effect of probiotics on liver weight are controversial [38]. It has been suggested in previous reports that liver weight is increased by the addition of probiotics [39]. However, our findings are different from previous reports. In our study, the liver yield was significantly reduced by LPJZ-658 supplementation for broilers. Probiotics have a lipid-lowering function. The effect of the use of probiotics was investigated in a previous report. The amount of abdominal fat deposition was significantly reduced by feeding broilers probiotics [34,40]. However, it was shown in the current study that there was no effect of LPJZ-658 on the abdominal fat deposition and intermuscular fat width of broilers.

Better chicken meat characteristics could contribute to extending the meat’s shelf life during storage and improving its taste. The meat color, pH value, CL, DL, and shear force are widely employed indices for assessing meat quality [41]. Postmortem pH reduction is critical for converting muscle glycogen to lactic acid because it influences meat color, texture, and water-holding capacity. It has been shown in a previous study that probiotics, as dietary supplements, could increase the pH_24h_ of BM [42]. In this study, the addition of LPJZ-658 improved the pH 24 h of BM and TM after the slaughter of broiler chickens. It has been reported in studies that dietary supplementation of probiotics or synbiotics did not alter meat color [33,43,44]. On the contrary, there was a change in meat color in both BM and TM with supplementation with LPJZ-658 in our study. The water-holding capacity is essential for whole meat and other processed meat and poultry products. This capacity can affect the functions and sensory properties of meat, which are related to texture, juiciness, flavor, and nutrition, as some nutrients may be lost during water exudation [45]. It was found that the addition of LPJZ-658 reduced the CL of the BM after storage for 24 h, indicating that LPJZ-658 had a promoting effect on the water retention of meat, consistent with the findings of similar probiotics studies on supplementation with Bacillus subtilis [33] or *L. plantarum* [46], and decreased muscle CL. Lipid peroxidation has been shown to deteriorate the quality of poultry meat, which decreases nutritional values, resulting in texture and flavor problems and altering meat appearance [47]. The amount of MDA and SOD reflect the degree of redox status of muscle [48]. Previous studies indicate that symbiotic supplementation decreased BM MDA content [43]. It was also reported that probiotics decreased serum MDA concentration and improved the activity of SOD [49]. However, in the current study, no effects of LPJZ-658 supplementation were observed on the MDA accumulation and SOD activity in both BM and TM (Supplementary Table S1). The above results showed that adding LPJZ-658 to the diet could improve the meat quality of broiler chickens but has no effect on the oxidative stress.

Serum immunoglobulins are typical parameters used to estimate the immune status of livestock. The effect of probiotics on the immune parameters and responses of broilers may be varied due to different probiotic strains [50,51,52]. It was reported in other studies that the levels of serum IgG were increased by dietary probiotics [53,54,55]. In this study, LPJZ-658 treatment can maintain the serum IgG concentration of broiler chickens at a high level (Table S2).

The small intestine is the leading site for nutrient absorption, whereas the villus can also play an essential role in absorbing nutrients. The villi length (VL)/CD and VH/CD in piglet and broiler chickens have been increased using several probiotics [56,57]. Other studies reported that dietary supplementation of *B. subtilis* remarkably decreased CD and elevated the VL/CD ratio in the duodenum of broilers [53,58]. Therefore, we further analyzed the morphologic alterations of the small intestinal of broilers. It was shown in the results that the addition of LPJZ-658 promoted the extension of duodenal villus height in broilers. We also found that the ileum villus height was increased by LPJZ-658 supplementation, while improving the VH/CD in broilers. Furthermore, our results are in agreement with the findings from weaned piglets [51,59] and broilers [32], in which it was shown that longer ileal villi could be promoted by *Lactobacillus rhamnosus* GG. Longer villi and a higher VH/CD ratio result in more absorptive epithelial cells and greater villi surface area, thereby promoting better nutrient absorption. This could explain the enhanced growth performance of the LPJZ-658-fed broilers.

The cecum is the most important intestinal organ in broilers, within which the microbiota is involved in regulating key host metabolic and immunologic health functions, including nutrient digestion and absorption, maintaining energy balance, and immune system development. The bacterial species richness is associated with the stability of intestinal microecology [60]. Therefore, we profiled the response of cecal microbiota composition to dietary LPJZ-658 supplementation. A variety of experiments showed that there were no differences in alpha diversity in the microbiota of broilers supplemented with probiotics in their diet [27,61,62]. Conversely, in the present study, dietary supplementation with LPJZ-658 raised alpha diversity remarkably. In addition, in the current study, the beta diversity index suggested a very good separation between the LPJZ-658 group and CON group. This finding is consistent with those of Zeng et al. [27]. Overall, the diversity of the cecum microbial community was affected by dietary LPJZ-658 supplementation. In addition, there were differences in the relative abundance of individual phyla and genera in this study. At the phylum level, we found that the most abundant phyla were Firmicutes and Bacteroidetes, but no significant difference was observed among two groups. The relative abundances of Proteobacteria, Actinobacteria, Verrucomicrobiota, and Acidobacteriota were significantly higher in the LPJZ-658 group. It is well-known that most of the members of Proteobacteria and Actinobacteria are enteropathogenic, which has been correlated with the pro-inflammatory cytokine profile of the chicken [63,64]. Interestingly, in the current study, elevations in the phyla Proteobacteria and Actinobacteria were observed after LPJZ-658 supplementation, which was inconsistent with earlier findings [65,66]. We speculate that this may be attributed to the difference in breeding environment and the complex composition and diverse effects of LPJZ-658 on intestinal microbiota. When the sequences were classified further into the genus levels, we found that the relative abundances of Streptococcus, Veillonella, Neisseria, and Haemophilus were significantly decreased in the LPJZ-658 group, and they were potentially harmful bacteria [67,68]. In contrast, there was an increase in the abundance of potentially beneficial bacteria (Bacteroides, Phascolarctobacterium, Bacillus, and Akkermansia) after LPJZ-658 supplementation. Bacteroides especially can provide nutrition for other microbial residents and enhance the body’s immunity [69,70]. Overall, the results suggested that dietary supplementation with LPJZ-658 is a potentially practical and effective strategy for improving the microbial community of broilers.

## 5. Conclusions

Our study shows that the addition of *L. plantarum* LPJZ-658, a newly discovered natural probiotic strain, to diets has positive effects on the growth production, meat quality, and intestinal morphology of broilers and changed the composition of their cecal microbiota. Therefore, we suggest LPJZ-658 supplements as an ideal alternative to antibiotics to improve broiler productivity.

## Figures and Tables

**Table 1:** Feed ingredients and nutrient content of the basal diets (%).

Ingredients	Basal Diet
Corn	59.32
Soybean meal	29.02
Soybean oil	3.01
Corn gluten meal 56%	2.00
Corn DDGS 28%	2.00
Calcium hydrogen phosphate	1.87
Limestone powder	0.92
L-lysine sulfate	0.60
DL-methionine	0.34
Salt	0.25
Mineral mix ^1^	0.20
Choline-chloride (50%)	0.15
Sodium bicarbonate	0.14
Threonine	0.12
Multi-vitamins ^2^	0.03
Phytase1000	0.02
Antioxidant	0.01
Total	100
Calculated nutrients, %	
Avian metabolic energy, MC/kg	3.00
Crude protein	20.00
Calcium	0.90
Total phosphorus	0.67
Non-phytate P	0.45
Salt	0.31
Lysine	1.30
Methionine	0.65
M. + Cystine	0.96
Threonine	0.85
Tryptophan	0.22
Sodium	0.18
Chlorine	0.18
Potassium	0.80
Crude fiber	3.00
Crude ash	5.33

Content by kg of diets: ^1^ Mineral mix: Copper = 12 mg; Iron = 110 mg; Zinc = 80 mg; Manganese = 100 mg; Iodine = 0.8 mg; Selenium = 0.4 mg. ^2^ Multi-vitamins: Vit. A = 12,500 IU; Vit. D = 6250 IU; Vit. E = 20 IU; Vit. K = 2.75 mg; Vit. B1 = 2.5 mg; Vit. B2 = 6.25 mg; Vit. B6 = 4 mg; Vit. B12 = 0.02125 mg; Calcium pantothenate = 8.75 mg; Nicotinamide = 32.5 mg; Folic acid = 1.075 mg; Biotin = 0.1125 mg.

**Table 2:** Growth performance in broilers at 42 days of age.

Items	CON	LPJZ-658	*p*-Value
ADG (g)	56.81 ± 1.80	65.02 ± 1.56	0.003
ADFI (g)	99.98 ± 3.17	109.23 ± 2.62	0.037
FCR	1.76 ± 0.02	1.68 ± 0.01	0.002

ADG, average daily gain; ADFI, average daily feed intake; FCR, feed conversion ratio. SEM: standard error of mean. Data are expressed as the means ± SEM. *p* < 0.05 was taken to indicate statistical significance.

**Table 3:** Carcass characteristics and immune organ index in broilers at 42 days of age.

Items	CON	LPJZ-658	*p*-Value
Carcass Characteristics			
Carcass yield (%)	94.18 ± 0.43	93.28 ± 0.48	0.178
Half-Eviscerated yield (%)	84.01 ± 0.45	83.02 ± 0.47	0.147
Eviscerated yield (%)	76.14 ± 0.58	75.39 ± 0.45	0.320
Abdominal fat yield (%)	1.12 ± 0.10	1.18 ± 0.08	0.664
Liver yield (%)	2.11 ± 0.06	1.81 ± 0.04	0.001
Breast muscle yield (%)	28.50 ± 0.10	28.89 ± 0.56	0.736
Thigh muscle yield (%)	19.15 ± 0.10	20.88 ± 0.34	0.004
Subcutaneous fat thickness (cm)	1.99 ± 0.17	1.67 ± 0.13	0.157
Intermuscular fat width (mm)	4.03 ± 0.26	3.59 ± 0.19	0.191
Immune Organ Index			
Thymus yield (%)	0.32 ± 0.03	0.33 ± 0.03	0.803
Spleen yield (%)	0.12 ± 0.01	0.13 ± 0.01	0.372
Bursa of fabricius yield (%)	0.19 ± 0.01	0.19 ± 0.01	0.927

SEM: standard error of mean. Data are expressed as the means ± SEM. *p* < 0.05 was taken to indicate statistical significance.

**Table 4:** Muscle quality in broilers at 42 days of age.

Items	CON	LPJZ-658	*p*-Value
Breast			
pH_45min_	6.39 ± 0.04	6.38 ± 0.06	0.825
pH_24h_	5.46 ± 0.05	5.79 ± 0.04	0.025
Meat color_45min_	72.86 ± 1.38	74.61 ± 1.29	0.367
Meat color_24h_	60.46 ± 0.99	67.45 ± 1.78	0.003
Drip loss_24h_ (%)	3.73 ± 0.21	4.23 ± 0.18	0.092
Drip loss_48h_ (%)	5.54 ± 0.33	5.48 ± 0.27	0.892
Cooking loss (%)	19.97 ± 0.87	15.67 ± 0.40	0.0003
Shear force (N)	24.20 ± 2.38	31.32 ± 3.60	0.116
Thigh			
pH_45min_	6.33 ± 0.06	6.27 ± 0.06	0.500
pH_24h_	5.77 ± 0.04	5.96 ± 0.06	0.026
Meat color_45min_	60.75 ± 0.55	67.81 ± 1.40	0.0002
Meat color_24h_	57.46 ± 1.75	64.11 ± 0.99	0.004

pH_45min_: muscle pH value at 45 min postmortem; pH_24h_: muscle pH value at 24 h postmortem; drip loss: drip loss of muscle after hanging at 4 °C for 24 h or 48 h; cooking loss: weight loss of muscle during heat treatment in a water bath at 75 °C for 20 min at 24 h postmortem after storage at 4 °C; meat color value measured at 45 min or 24 h postmortem. SEM: standard error of mean. Data are expressed as the means ± SEM. *p* < 0.05 was taken to indicate statistical significance.

**Table 5:** Intestinal development of broilers at 42 days of age.

Length (cm)	CON	LPJZ-658	*p*-Value
Duodenum	28.80 ± 1.64	27.4 ± 0.65	0.438
Jejunum	67.10 ± 2.72	73.00 ± 1.86	0.091
Ileum	64.70 ± 2.14	72.10 ± 2.37	0.032
Cecum	16.20 ± 0.43	18.56 ± 0.77	0.015

**Table 6:** The small intestine histomorphology of broilers at 42 days of age.

Items	CON	LPJZ-658	*p*-Value
Duodenum			
Villus height (μm)	1022.00 ± 102.5	1483.00 ± 42.36	0.001
Crypt depth (μm)	265.00 ± 42.48	320.40 ± 21.18	0.231
VH/CD (μm)	3.98 ± 0.35	4.70 ± 0.22	0.101
Jejunum			
Villus height (μm)	994.30 ± 9.52	1118.00 ± 95.43	0.232
Crypt depth (μm)	204.50 ± 14.11	181.20 ± 13.18	0.261
VH/CD (μm)	4.97 ± 0.42	6.24 ± 0.49	0.084
Ileum			
Villus height (μm)	618.30 ± 36.89	865.80 ± 12.86	0.0002
Crypt depth (μm)	163.40 ± 22.12	169.20 ± 8.17	0.813
VH/CD (μm)	3.96 ± 0.33	5.15 ± 0.18	0.014

SEM: standard error of mean. Data are expressed as the means ± SEM. *p* < 0.05 was taken to indicate statistical significance.

## Data Availability

The data are shown in the main manuscript.

## Supplementary Materials

The following supporting information can be downloaded at: https://www.mdpi.com/article/10.3390/microorganisms11061549/s1, Table S1:Muscular antioxidant status in broilers at 42 days of age; Table S2: The levels of immunoglobulins in the plasma of broilers at 42 days of age.
