# Supplementary material for: Effects of Lactiplantibacillus plantarum LPJZ-658 Supplementation on the Production, Meat Quality, Intestinal Morphology, and Cecal Microbiota of Broilers Chickens
Source: Microorganisms. 2023 Jun 10;11(6):1549. doi: 10.3390/microorganisms11061549 (PMC10301381; doi:10.3390/microorganisms11061549)
Supplement: Supplementary file 1 [file microorganisms-11-01549-s001.zip › Supplementary Table.pdf]

Table S1. Muscular antioxidant status in broilers at 42 days of age

Items	CON	LPJZ-658	*p*-Value
Breast			
MDA (nmol mg prot^{-1})	1.34 ± 0.02	0.21 ± 0.04	0.072
SOD (U mg prot^{-1})	24.19 ± 2.71	22.62 ± 4.25	0.760
Thigh			
MDA (nmol mg prot^{-1})	1.73 ± 0.25	1.49 ± 0.25	0.452
SOD (U mg prot^{-1})	50.01 ± 4.91	56.75 ± 8.23	0.493

MDA, malonaldehyde; SOD, superoxide dismutase. SEM: standard error of mean. Data are expressed as the means ± SEM. $p < 0.05$ were taken to indicate statistical significance.

Table S2. The levels of immunoglobulins in the plasma of broilers at 42 days of age

Items	CON	LPJZ-658	*p*-Value
IgM (mg/ml)	31.02±2.79	32.54±2.48	0.689
IgG (mg/ml)	18.63±0.93	27.21±2.80	0.015
IgA (mg/ml)	1.28±0.11	1.3±0.06	0.856

SEM: standard error of mean. Data are expressed as the means ± SEM. $p < 0.05$ were taken to indicate statistical significance.
